# Seasonality of Suicidal Behavior

**DOI:** 10.3390/ijerph9020531

**Published:** 2012-02-14

**Authors:** Jong-Min Woo, Olaoluwa Okusaga, Teodor T. Postolache

**Affiliations:** 1 Department of Psychiatry, Seoul Paik Hospital, Inje University School of Medicine, Mareunnae-ro 9, Jung-gu, Seoul, 100032, Korea; Email: jongmin.woo@gmail.com; 2 Stress Research Institute, Inje University, 607 Obang-dong, Gimhae, Gyungnam, 621749, Korea; 3 Psychiatry Residency Training Program, St. Elizabeth’s Hospital, 1100 Alabama Avenue, Washington, DC 20032, USA; Email: olaokusaga1@yahoo.com; 4 Mood and Anxiety Program (MAP), Department of Psychiatry, School of Medicine, University of Maryland, Baltimore, MD 21201, USA

**Keywords:** seasonality, suicide, prevention

## Abstract

A seasonal suicide peak in spring is highly replicated, but its specific cause is unknown. We reviewed the literature on suicide risk factors which can be associated with seasonal variation of suicide rates, assessing published articles from 1979 to 2011. Such risk factors include environmental determinants, including physical, chemical, and biological factors. We also summarized the influence of potential demographic and clinical characteristics such as age, gender, month of birth, socioeconomic status, methods of prior suicide attempt, and comorbid psychiatric and medical diseases. Comprehensive evaluation of risk factors which could be linked to the seasonal variation in suicide is important, not only to identify the major driving force for the seasonality of suicide, but also could lead to better suicide prevention in general.

## 1. Introduction

Several epidemiological studies have described a seasonal variation of suicide rates, with a highly replicated suicide peak in springtime [1,2]. However, recent studies have shown that the amplitude of the spring peak is on the decline, while new small peaks are occurring at other times of the year, especially in industrialized Western countries [3,4]. In spite of it being a well replicated phenomenon, the empirical finding of seasonal peaks in suicide is poorly understood. 

To date many risk factors for suicide have been reported and they can be categorized by demographic, social and clinical characteristics. Such risk factors include age, gender, rural/urban area of residence [5], race [6], month of birth [7], socioeconomic factors [8], marital status [9], inter-personal relationships or life events [10,11], comorbid medical conditions, current or history of psychiatric illness [12], allergy [13], and most importantly, previous suicide attempts and violent methods of prior suicide attempt [2,14]. Physical environmental factors, e.g., sunshine, temperature [15], chemical (e.g., air pollutants) [16] and biological factors such as viruses [17], parasites such as *Toxoplasma gondii*, and aeroallergens [18,19] have also been associated with suicide risk. 

Among the numerous risk factors for suicide, it is important to define those that are fluctuating, modifiable, and potentially treatable. Since the seasonal fluctuation in suicide has become a recognized and significant phenomenon, it is desirable to identify variables that consistently demonstrate an association with the seasonal variation of suicidal behaviors as well as completed suicide. For example, environmental factors such as the amount of sunshine and distribution of aeroallergens vary with the seasons. Moreover, clinical variables such as allergic illness, viral infections and mood disorders also manifest seasonal variations and such variations could potentially be associated with the seasonal variation of suicide rate. 

A better understanding of the underlying mechanisms responsible for the seasonal variations in suicide could lead to improved and novel suicide prevention strategies. Therefore we comprehensively evaluated published papers, focusing on the potential association between suicide risk factors and seasonal fluctuation of suicide completion in various demographic groups and geographic locations. We also discuss the presence of seasonality of suicide, the strength and the clinical implication of the association for each risk factor. 

## 2. Methods

This is a comprehensive narrative review of journal papers on suicide seasonality published from 1979 to 2011. We carried out a comprehensive search of PubMed/MEDLINE (1979–2011) using the keywords: “suicide” and “seasonality”, cross-referenced with the terms “age”, “gender”, “methods of suicide”, “socioeconomic status”, “sunshine”, “temperature”, “geographic region”, “comorbid disease”, “allergy”, “mental illness”, “infection”, and “cytokine”. After we had identified potential publications of interest we read through the titles and abstracts and those selected were subsequently reviewed and categorized by suicide risk factors of interest. We only included articles in English. Among those, reports dealing with seasonality or monthly fluctuation were taken to review the relationship between seasonality and suicide.

## 3. Potential Environmental Mediators

Environmental factors have been considered as possible mediators of the seasonal variation in human behaviors and therefore may also influence suicidal behaviors. Here we review physical (*i.e.*, bioclimatic factors such as sunshine, temperature and rainfall), chemical (*i.e.*, pollutants), and biological (*i.e.*, viruses, bacteria, protozoa and allergens) factors as potential triggers of suicidal behaviors in spring or fall. 

### 3.1. Bioclimatic Factors

Bioclimatic factors have been suggested to be potential mediators of the seasonal variation in suicide, though this concept is controversial. Some researchers have documented a positive association between sunshine/temperature/humidity and suicide [20,21,22,23,24,25,26], while others dispute this relationship [27,28,29,30]. In addition, a few studies concluded that a positive association between climatic factors and seasonal variation of suicide was present only for suicide by violent methods [14,31]. 

Petriduo *et al*. [32] suggested that sunlight may act as a trigger of suicide. In addition, suicide rates are greater in rural areas than in urban areas [2,4,33,34] and higher among outdoor workers compared to indoor workers [35]. Some empirical findings support the notion that the intensity of sunlight may play a role in the triggering of suicide and therefore provide a potential link to the seasonal variation in suicide. Hiltunen *et al*. reported the association between increased suicide mortality and the period with the longest day length (which was between May and July) *i.e.*, late spring/early summer [36]. Another study in Greenland reported a similar pattern. However, both studies suggested the role of latitude and other signals besides the variation in daylight, as the suicide peak of the northern area of Finland (Oulu) was delayed when compared to the southern area (Helsinki) and the strength of the suicide peak was more pronounced at higher latitudes [36,37]. A recent analysis of data from Finland suggested a correlation between solar radiation and suicide mortality [38] but other studies have also suggested that seasonal suicide peak in spring occurs significantly later than the interval of change in day length [39,40,41]. Furthermore, Papadopoulos *et al*. [42] hypothesized that a time lag exists for the effect of solar radiance on suicidality. In summary, seasonal changes in sunlight seem unlikely to fully account for the seasonal variation in suicide. 

With regard to temperature, a study performed by Volpe *et al*. [25] found that suicide rates in Brazil not only showed a higher peak in December and January than the rest of the year, but were also significantly correlated with increasing temperature. In addition, Kim *et al*. [15] reported a 1.4% increase in suicide when temperature goes up by 1 degree Celsius. Temperature could either be a marker of seasonal change, or the mediator of it. In addition, specific meteorological conditions such as temperature and thunderstorm for the preceding day could contribute to increased risk of suicide in individuals [26]. 

Precipitation (rainfall and snowfall) is another climatic factor that shows seasonal variation and has thus been postulated to possibly be predictively associated with seasonality of suicide. When Ajdacic-Gross *et al*. [28] modeled monthly data on suicide and precipitation in Switzerland precipitation did not show any noteworthy effects on suicide frequencies. Lin *et al*. also examined the association between monthly suicide rates and climatic influences including atmospheric pressure, temperature, sunshine, humidity, and rainfall in Taiwan; however, they only found evidence of an association of temperature with seasonality, but reported no significant association between rainfall and seasonal peaks of suicide in spring/early summer [43].

### 3.2. Geographic Location

Chew and McCleary [4] comprehensively compared the seasonal variation of suicide across 28 nations and found well replicated seasonal spring peaks in suicide rates from the various nations regardless of the location of the countries. They also observed wide cross-sectional variation in degree of suicide seasonality. For instance, when comparing Canada to Portugal they demonstrated a narrow range of seasonal fluctuation in Canada (ratio of average spring to average winter = 1.08) *versus* wide fluctuation of seasonal suicide rates in Portugal (ratio of average spring to average winter = 1.70) implicating a more prominent seasonal spring peak in Portugal.

Consistent with the pattern in the northern hemisphere, Flisher *et al*. [44] reported a mirror image spring or summer peak of suicide and a trough in fall in South Africa, especially for less urbanized subpopulations. Similarly, studies in Australia [24] are concordant with studies conducted in the Northern Hemisphere in Europe [1,10,11,32,45,46,47,48] and Asia [18,49,50], identifying a seasonal spring suicide peak. 

### 3.3. Allergens

Allergy has been previously linked to suicide [13]. The seasonality of suicide has been shown to co-occur with the seasonal peaks in ambient pollen concentration during spring (*i.e.*, tree pollen), summer (*i.e.*, grass pollen), and fall (*i.e.*, ragweed) [19]. Pollens are aeroallergens and are capable of inducing an allergic inflammatory reaction when they reach the intranasal mucosa of susceptible individuals. The inflammatory reaction induced by aeroallergens involves the production of Th2 cytokines which, in animal models, have been associated with increased anxiety-like behavior, reduced social interaction [51] and aggressive behavior [52] all of which can be considered as endophenotypes for suicidal behavior [53]. Furthermore, the seasonal peak in aeroallergens resulting in the concomitant worsening of allergy symptoms could (via inflammatory mediators of worsening allergy symptoms) potentially worsens depressive symptoms, anxiety and impulsivity in mood disorder patients, resulting in exacerbated risk of suicidal behavior. Consistent with this notion, Manalai *et al*. [54] recently reported that in bipolar patients pollen-specific IgE positivity and worsening of allergy symptoms are associated with worsening of depression scores during exposure to aeroallergens. In addition, changes in allergy and anxiety (anxiety representing a potentially independent suicide risk factor) in patients with mood disorders exposed to seasonal peaks of aeroallergens were observed to be correlated [54]. In essence, the current available evidence makes seasonal fluctuation of aeroallergens a possible factor involved in the underlying mechanisms responsible for seasonality of suicide. This is particularly important from a neuroimmune perspective, considering a previous study showed an increased gene expression for cytokines involved in allergic reactions in the orbitofrontal cortex (a region previously implicated histopathologically with suicide) in victims of suicide [53].

### 3.4. Viruses

The human immunodeficiency virus (HIV) has been associated with suicide [55] but no seasonal pattern has been reported in relation to HIV-related suicide rates neither has HIV been known to manifest a seasonal pattern of infectivity. The influenza virus, on the other hand, has a seasonal pattern of infectivity. However the only report of an association of influenza with suicidal behavior [17] did not include an evaluation of seasonality effect on suicide. More studies on the association of seasonal viruses and suicide are needed. 

### 3.5. Pollutants

Air pollutants have been correlated with rates of visits to the emergency room as well as inpatient admission rates of patients with mental illness [56]. Recently Kim *et al*. [16] found that the rate of completed suicide in the Republic of Korea was elevated when there was an increase in the ambient particulate matter two days prior to the day of suicide. The two aforementioned studies did not take into consideration the impact of the season on elevation of suicide risk. Szyszkowicz [57], however, carried out an analysis of data on air pollution effect on emergency room (ER) visit for worsening depression by season and found that the highest percentage of depression-related ER visits were during periods of increased concentration of ambient particulate matter during the cold season and the finding was limited to only females. However, the findings by Szyszkowicz should be interpreted with caution (in terms of elucidation of the seasonality effects of air pollutants on depression), since the analysis did not include an assessment of an interaction between season and pollutant but rather an analysis by season was carried out. It therefore appears that the literature on the potential contribution of pollutants to the seasonality of suicide is sparse and no generalization can be made at this time. 

## 4. Clinical Determinants: Effect of Morbidity

It has been well described that psychiatric disorders are associated with suicide and at the time of suicide completion, more than 90% of suicide victims suffer from a psychiatric disorder [58]. Reports from Finland showed an association between time patterns of attempted suicides and psychiatric disorders (e.g., mood disorders, substance use disorders and schizophrenia-related disorders [59,60]. 

**Figure 1:**
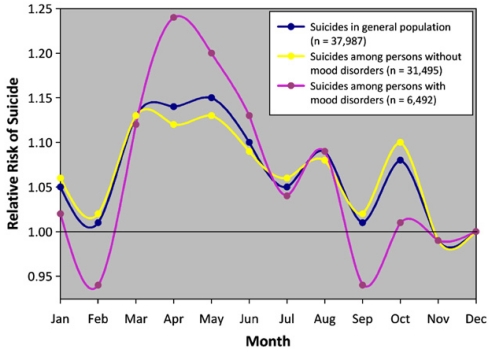
Monthly rates of suicide based upon all cases of suicide from 1970 to 2001in the Danish Cause of Death Registry. History of mood disorder was obtained from the Danish Psychiatric Central Register. The authors estimated the monthly rate ratio of suicide relative to December. Conditional logistic regression analyses with adjustment for place of residence, marital status, income, and method of suicide were used to compare seasonality of suicide in victims with *versus* without hospitalization for mood disorders. A statistically significant spring peak in suicide was observed in both groups. A history of mood disorders increased the risk of suicide in spring (for males: RR = 1.18, 95% CI 1.07–1.31; for females: RR = 1.20, 95% CI 1.10–1.32). Reproduced from [19] with permission.

However, studies on the relationship between seasonal variation in the occurrence or exacerbation of mental disorders and suicide seasonal peaks are limited [45,61,62]. A study [61] conducted in Sweden showed a seasonal spring/early summer peaks among patients diagnosed with neurotic, stress-related, or somatoform disorder; however, only patients with symptoms severe enough to require hospitalization were studied. Consistent with this study, Brådvik *et al*. [62] demonstrated a seasonal spring peak of suicide in a study of male patients with alcohol addiction. Rocchi *et al*. [45] also reported on the seasonality of suicide completion among patients with psychiatric illnesses. Recently, Postolache *et al*. [63] reported an increased amplitude of the suicide peak in spring among victims of suicide with a history of mood disorders (see Figure 1). Another study carried out by Kim *et al*. [64] demonstrated seasonal spring/summer peak of suicide completion in depression and fall/winter peak in schizophrenia. 

In addition, significant seasonal peaks were reported in allergy-related asthma, rhinitis, and atopic dermatitis. As allergy-related diseases are associated with suicide completion, seasonal changes in allergens may lead to seasonal increase in incidence and exacerbation of allergic disorders which in turn could potentially be associated with peak in suicide rates, mediated through molecular and cellular components of allergic inflammation affecting the brain [13]. Indeed, Timonen *et al*. [65] revealed an association between prior hospitalization for atopic disorders and seasonal variation of suicide.

## 5. Demographic Variables

### 5.1. Age

Several studies have found associations between age and seasonal variation in suicide. Maes *et al*. [2] reported that the suicide rate among younger people was increased in spring (*i.e.*, March and April), whereas the rate within older adults was raised in late summer (*i.e.*, August). However, Lahti *et al*. [66] observed a suicide peak in fall among adolescents, particularly for those dying by shooting. Furthermore, McCleary *et al*. [67] documented that a suicide peak was observed in younger aged individuals in winter and fall, while suicide among the very old was elevated towards the summer period. The inconsistency between studies may reflect methodological or environmental differences between studies and at the moment, no conclusion can be drawn.

### 5.2. Gender

Although seasonality of suicide completion is seen both in men and women, the seasonal patterns differ between genders. For example, only a single spring peak is found in men, while two peaks in spring and fall have been reported in women [4,33,34,40,68]. In England, middle aged women who had school-aged children were more likely to commit suicide in fall, which was the beginning of the school year [4,69]. Though speculative, it may be that a sudden reduction in the duration of direct contact with a dependent represents a type of suicide risk in these women. Gender effect on seasonality of suicide was also noted in Hungary where a steadily increasing prescription rate for antidepressants was associated with a decrease in national suicide rate but significantly decreased seasonality of suicide only in males [70]. The suicide peak in spring has been considered to be a consequence of seasonal occurrence of depression-related suicides and the decreased seasonality of suicide in this Hungarian study was suggested to be a marker of lowering depression-related suicides (especially among men) as a result of increased antidepressant utilization in the population [70].

Seasonality of suicide attempts is also shown to be associated with gender [71]. Studies performed in Scotland and in Oxford revealed a seasonal variation of female suicide attempts with increased rates during summer and decreased rates in winter, but no significant seasonal variation of male suicide attempts was found [72,73]. In addition, the results of the WHO/EURO Multicenter study on Parasuicides indicated that the seasonal pattern of suicide attempts in women showed a peak in spring and nadir in winter, but no significant variation of suicide attempts was observed within the male subpopulation [74]. However, there have been negative reports as well. Mergl *et al*. analyzed suicide attempts in Nuremberg and Wuerzburg from 2000 to 2004 and they failed to confirm the significant gender difference in seasonality of suicide attempts [71]. Kreitman *et al*. also reported no considerable gender difference in seasonality of suicide attempts in the U.S. [75].

### 5.3. Month of Birth

While several studies have reported season of birth effect on suicide or suicidal behavior [7,76,77,78], there is lack of evidence to indicate an association between month of birth and seasonality of suicide completion. Dome *et al*. [76] found a significantly increased risk of suicide completion among those individuals who were born in spring and summer, however, this study did not show any relationship between season of birth and seasonal variation of suicide. Another study which evaluated the effect of birth month on suicidal behavior in Western Australia reported a notable spring peak of deliberate self-harm and a significantly increased birth in spring within the deliberate self-harm group [7]. However, no season-of-birth effect was observed in relation to completed suicide in the study. 

### 5.4. Socioeconomic Factors

Socioeconomic status can affect suicide rates. Social discrepancy, disputes, socio-economic gradient (urban-rural income gradient, *etc*.), divorce and resulting single parent family environment can be related with seasonality of suicide. The majority of research findings indicated that the seasonal spring peaks are greater in rural areas compared to urban areas [2,4,79]. In particular, Micciolo *et al*. [79] evaluated the seasonality of suicide in Italy from 1969 to 1984 and found the suicide peaks in spring to be more notable in rural areas than in urban areas, although the suicide rates was higher in urban regions. A review by Christodoulou *et al*. [80] suggested that this phenomenon might plausibly be related to intensity of seasonal activities such as agricultural work in the rural areas. In fact, Chew and McCleary [4] reported that the spring peak of suicide is relatively larger in agricultural countries compared to industrial countries. They also found that the larger amount of agricultural work is significantly correlated with the greater spring peak of suicide. Ajdacic-Gross *et al*. [81] further posited that as the traditional rural society is disappearing with industrialization, the seasonal variation of suicides might be attenuated. 

In addition, seasonality of suicide has been shown to be related to occupational differences. The agricultural and construction sectors usually have intense activity from spring to fall. Näyhä [40] found that suicide committed by people who served in technical, administrative, and service work (*i.e.*, modern occupations) usually peaked in late fall, while people who engaged in traditional occupations (e.g., agriculture, transport, or manufacturing work) showed seasonal peaks of suicide in spring/ summer. Koskinen *et al*. [35] also examined seasonality of suicide in different occupations including farmers, forest workers, construction, and indoor workers. They documented that spring peak and winter trough of suicide pattern was observed in groups of farmers and forest workers. On the contrary, a significant summer nadir was shown within indoor workers. Moreover, in their sub-group analyses by suicide methods, 90.5% of farmers used violent methods, followed by forest workers (79.1%), construction (73.2%), and indoor workers (69.2%), indicating violent suicides decreased among indoor workers. Considering suicides by violent methods show remarkable peaks in spring [2,43,47], it is plausible to expect seasonal spring peaks with people who are more likely to spend time in outdoor settings [82]. Migrant workers can be exposed to higher mental distress and suicide risk as dramatically depicted in the series of attempted or committed suicides in Foxconn production facilities in China between Jan and May 2010 [83,84,85]. However, seasonal variation of suicide in migrant populations needs to be further studied. 

These findings seem to indicate that people who are more exposed to the outdoor environment have a greater seasonal spring peak in suicide—thus, suggesting that factors driving seasonality may be more abundant in the outdoor environment. For example, increased seasonal work related-stress in farmers and increased exposure to outdoor physico-chemico-biological factors such as day length, light, temperature, pollution, pathogens or allergens may contribute to more ample seasonal suicide peaks. 

## 6. Suicide Methods

Suicide methods can be classified as either violent (*i.e.*, hanging, firearms, drowning, jumping, cutting, or self-immolation) or non-violent (*i.e.*, ingestion of poisons, drugs, gases, or vapors) in terms of lethality based on the International Classification of Diseases [80]. There appears to be seasonal variation of suicide completion by suicide methods. A number of researchers have reported seasonal variation of suicide by violent methods including hanging, jumping from a height, drowning, poisoning, and firearms [3,41,47,66,86,87]. Suicide rates by violent methods peak in spring/early summer and dip in winter, which is consistent with the general pattern of suicide seasonality. Hakko *et al*. [39] reported that suicide rates by violent methods increased by 16% in May, while it correspondingly decreased by 15% in December. The patterns of seasonal fluctuation in violent suicides are well replicated, regardless of geographical region. Studies conducted in Europe including Finland [39,88], Italy [47,68], Greece [48], Belgium [2,89], Greenland [37,90], Switzerland [1,81], UK [91], Australia, New Zealand [92], Asia [43], and the U.S. [93,94] found seasonal spring peaks in violent suicide rates. In Taiwan, however, the violent suicide peaks in summer rather than in spring [43]. 

Gender differences have been reported with the use of violent suicide methods. Lester and Frank analyzed a U.S. population-based data and reported seasonal spring peaks of suicide by poison, hanging, or firearms, in addition to seasonal autumn peaks for hanging or firearms among male victims [94]. In contrast, in female victims, they observed seasonal variation of suicide completion with spring and fall peaks by poison or hanging and with summer/late fall peaks by firearms. Furthermore, the study conducted by Yip *et al*. in which they evaluated Australia-New Zealand population based data, revealed a significant seasonal variation of suicide by hanging in Australian and New Zealand in males only [92].

Regarding non-violent methods, Hakko *et al*. [39] found two peaks of suicide rates within the non-violent subgroup approximately a 9% increase in spring and an 8% increase in fall. However, the majority of studies did not observe any significant seasonal spring peaks in suicides by non-violent methods [2,43,68,89]. Pollen counts have been particularly related to nonviolent suicides in women [19].

As one of the possible mechanisms to explain the significant spring peaks of violent suicides, we can consider the role of neurotransmitters in violent behaviors. For example, serotonin concentration, is often associated with impulsive and aggressive behaviors [95] and tryptophan (the main precursor of serotonin) concentration in the brain shows a prominent seasonal rhythm with lower plasma levels measured in spring in comparison to other seasons [96]. Thus, researchers have postulated that low levels of serotonin in the brain could possibly have an influence on impulsive drives, violent behaviors, and potentially result in an individual committing suicide by violent methods [68,96]. A counter argument against the proposition of serotonin mediation of violent suicide could stem from the findings from an Australian study in which hours of bright sunlight exposure were directly correlated with serotonin turnover in the brain, measured invasively [97]. Brain serotonin turnover was seven times higher during the summer than during the winter, thus not entirely consistent with a hypothesis of a serotonergic mediation of suicide seasonality (*i.e.*, low serotonin in spring). 

A number of researchers have argued that seasonal variation of suicide by specific methods was determined by the opportunities to access the methods [3]. Ajdacic-Gross *et al*. [3] reported that whereas firearms and knives are normally available during the whole year, poisoning (especially pesticides) occurred in the planting season and drowning and jumping are mostly used in outdoor activity season. Lahti *et al*. [66] found that suicide by shooting among Finnish adolescents occurred more frequently from August to October and its monthly pattern was positively related to the duration of daily sunshine hours, which were suggested to be related to increased firearm availability during the hunting season in addition to other psychosocial factors. 

Seasonality of suicide by methods can vary across different time frames. Ajdacic-Gross *et al*. [81] looked at 120 year trends of suicide seasonality in Switzerland and determined that there was a decline of overall seasonal variation during 1969–2000 compared to 1881–1920. The most significant difference between the two periods was the attenuation of suicides by hanging and drowning, both of which previously had strong seasonal effects on suicide. Although statistically significant seasonal peaks in spring were exhibited in both periods, the strength of the association has been on the decline with regards to hanging. 

## 7. Conclusions

**Figure 2:**
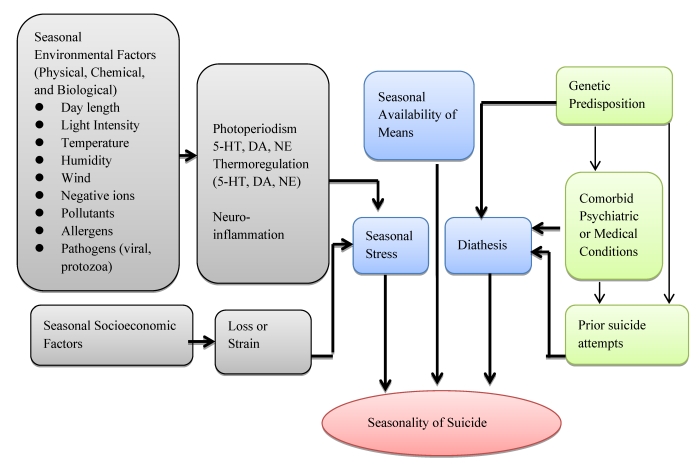
A Model depicting the interplay of factors potentially involved in the Seasonality of Suicide. 5-HT = 5-hydroxytryptamine; DA = dopamine; NE = norepinephrine.

Seasonal variation of suicide rates with the most common peak occurring in late spring or summer are one of the most consistent themes from environment-suicide research. In contrast, interactions between demographic factors, environmental factors and suicide methods have yielded inconsistent results. We synthesized Figure 2 to integrate variables related with seasonality of suicide.

Although the seasonal patterns of suicidal behavior are highly replicated, the underlying mechanisms are poorly understood and efforts to isolate seasonal variables, such as bioclimatic and socioeconomic variables, to assist in identifying factors mediating seasonality have often resulted in inconclusive findings. 

One of the explanations for this inconsistency could be differences in methodology across studies [29]. Obviously, all seasonality-suicide studies are inherently based on correlational studies which cannot explain causal relationships It is desirable to collect data spanning several years and including people from multiple geographical regions to avoid confounding effects from non-seasonality variables and over-generalization bias [26]. Unfortunately, many studies assessed seasonality over a relatively short period of time with data gathered in only one country or even smaller geographic unit [29]. Also, it will be important to establish a consistent set of multilevel variables all studies must account for when analyzing seasonal effects. For instance, in our recent study, after adjusting for the density of psychiatrists, urban *vs*. rural location and income, significant relationships between airborne allergens and suicide across space have been lost, suggesting a spurious relationship [98]. 

In some countries, seasonal suicide peaks have a tendency of being flattened in terms of reduced amplitude and smaller proportion of variance accounted for by the season. Recent studies using data from England and Wales [99], Hong Kong [100], Sweden [101], and Denmark [102] have demonstrated a diminishing seasonality tendency on suicides. However, this phenomenon does not apply in some other countries, such as Finland [20,39,41] and the United States [103], where a resilient seasonality pattern continues to be found for suicides or parasuicides. Overall, there might be a possibility that the contribution of season, while present, is so small that it can be irrelevant when other risk factors, such as gender and mental illnesses, are adjusted for. Few studies have examined seasonality in the context of other risk factors [104,105,106].

A better understanding of the mechanisms leading to seasonal peaks of suicide attempts and completions, may lead to identifying factors that could be amenable to preventative interventions and result, in the longer run, in flattening seasonal peaks of suicide and possibly, improved suicide prevention in general 
